# Stress-responsive regulation of extracellular proteostasis

**DOI:** 10.1083/jcb.202112104

**Published:** 2022-02-22

**Authors:** Jaleh S. Mesgarzadeh, Joel N. Buxbaum, R. Luke Wiseman

**Affiliations:** Department of Molecular Medicine, Scripps Research, La Jolla, CA

## Abstract

Genetic, environmental, and aging-related insults can promote the misfolding and subsequent aggregation of secreted proteins implicated in the pathogenesis of numerous diseases. This has led to considerable interest in understanding the molecular mechanisms responsible for regulating proteostasis in extracellular environments such as the blood and cerebrospinal fluid (CSF). Extracellular proteostasis is largely dictated by biological pathways comprising chaperones, folding enzymes, and degradation factors localized to the ER and extracellular space. These pathways limit the accumulation of nonnative, potentially aggregation-prone proteins in extracellular environments. Many reviews discuss the molecular mechanisms by which these pathways impact the conformational integrity of the secreted proteome. Here, we instead focus on describing the stress-responsive mechanisms responsible for adapting ER and extracellular proteostasis pathways to protect the secreted proteome from pathologic insults that challenge these environments. Further, we highlight new strategies to identify stress-responsive pathways involved in regulating extracellular proteostasis and describe the pathologic and therapeutic implications for these pathways in human disease.

## Teaser

Mesgarzadeh et al. describe the stress-responsive signaling pathways that regulate the activity and composition of the extracellular proteostasis network.

## Extracellular proteostasis in human disease

Nearly one-third of the human proteome is targeted to the ER for folding and trafficking to secretory environments such as the extracellular space. In extracellular environments such as the blood and cerebrospinal fluid (CSF), secreted proteins are involved in numerous and diverse biological functions including interorganellar metabolic regulation, trafficking of small-molecule hormones and proteins, structural support of tissues, coagulation, and immunological and neurological signaling (Wyatt et al., 2012, 2013). All these functions require secreted proteins to maintain a folded, functional conformation. However, harsh conditions in extracellular environments, including low ATP levels and high levels of oxidative and shear stress, challenge the ability of secreted proteins to maintain folded, functional conformations. Numerous human diseases are characterized by the toxic misfolding and aggregation of secreted proteins in extracellular environments. The most prominent is a subgroup of protein aggregation diseases commonly referred to as the amyloidoses that are caused by the misfolding and subsequent aggregation of a secreted amyloidogenic protein into toxic oligomers and structurally well-ordered amyloid fibrils (Blancas-Mejia and Ramirez-Alvarado, 2013; Wechalekar et al., 2016; Chiti and Dobson, 2017; Iadanza et al., 2018). 36 different proteins are known to deposit as amyloids associated with human diseases (Benson et al., 2020). These disorders are classified as systemic or localized based primarily on the site of amyloid deposition. The systemic amyloid diseases such as the transthyretin (TTR) amyloidoses and light chain amyloidosis are associated with the deposition of the amyloidogenic proteins TTR or amyloidogenic Ig light chains (LCs), respectively, in tissues distant from their site of synthesis (Benson et al., 2020). In contrast, local amyloid diseases such as Alzheimer’s disease (AD) and the prion disorders Creutzfeldt-Jakob disease and Gerstmann-Straussler syndrome involve the deposition of amyloid fibrils near or at their site of synthesis (Benson et al., 2020).

Despite the importance of extracellular protein aggregation in the pathogenesis of these disorders, the molecular factors that promote misfolding and aggregation of amyloidogenic proteins remain poorly defined. Intriguingly, genetic, environmental, and aging-related insults that challenge extracellular protein homeostasis (or proteostasis) have been implicated in the pathogenesis of numerous amyloid diseases. Destabilizing mutations in amyloidogenic proteins including TTR, lysozyme, cystatin C, gelsolin, and others predispose these proteins to misfold into aggregation-prone conformations, accelerating their concentration-dependent aggregation into toxic oligomers and amyloid fibrils (Blancas-Mejia and Ramirez-Alvarado, 2013; Chiti and Dobson, 2017; Iadanza et al., 2018). Chronic inflammation increases expression of the acute phase response protein serum amyloid A (SAA), the cleavage of which releases the amyloidogenic AA fragment, allowing its aggregation and subsequent deposition in the extracellular space (Brungers et al., 2020). Aging is also a risk factor for many amyloid diseases including AD (Frakes and Dillin, 2017; Sengoku, 2020). However, the importance of these types of insults in the toxic aggregation of secreted proteins suggests that deficiencies in the ability of organisms to regulate extracellular proteostasis is a critical determinant in the pathogenesis of these disorders. This has led to significant interest in understanding the molecular mechanisms that regulate proteostasis in extracellular environments.

**Figure 1.**
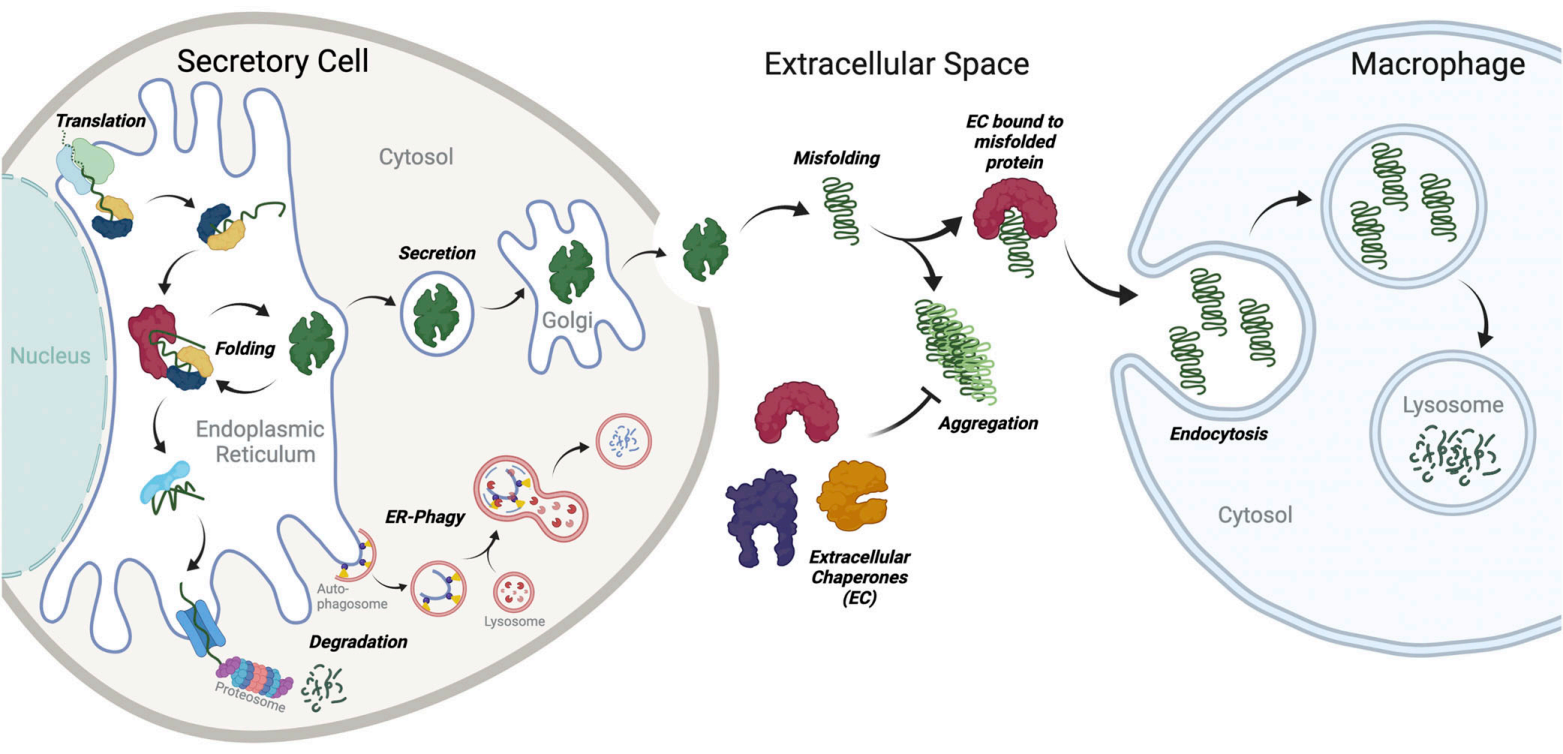
**ER proteostasis pathways and secreted chaperones coordinate to regulate extracellular proteostasis.** The integrity of the secreted proteome is regulated by the combined activity of proteostasis pathways localized to the ER and extracellular space. In the ER, quality control pathways partition secreted proteins between folding and degradation pathways to prevent secretion of nonnative and/or potentially aggregation-prone proteins to extracellular environments where they could aggregate into potentially toxic conformations. In the extracellular space, nonnative secreted proteins are bound by extracellular chaperones that prevent their aggregation and/or target these proteins to macrophages for endolysosomal degradation. Created with BioRender.com.

Extracellular proteostasis is primarily maintained through the activity of proteostasis pathways localized to the ER and extracellular space (Fig. 1). These pathways comprising chaperones, folding enzymes, and degradation factors engage nonnative proteins and facilitate their refolding and/or degradation in the early secretory pathway or extracellular environments (Wyatt et al., 2013; Plate and Wiseman, 2017; Sun and Brodsky, 2019; Chaplot et al., 2020; Romine and Wiseman, 2020; Satapathy and Wilson, 2021a; Satapathy and Wilson, 2021b). In response to stress, the composition and activity of ER and extracellular proteostasis pathways are regulated through the activity of multiple different stress-responsive signaling pathways. This provides a mechanism to protect the secreted proteome from the accumulation of nonnative or aggregation-prone proteins that can misfold and aggregate into toxic conformations during stress. Here, we discuss recent results highlighting the stress-responsive regulation of ER and extracellular proteostasis pathways, specifically focusing on the implications of this remodeling on the integrity and activity of the secreted proteome.

## ER-dependent regulation of extracellular proteostasis

### The integrity of the secretory proteome is regulated by ER quality control

The ER is the first organelle of the secretory pathway and is responsible for the folding and secretion of most secreted proteins, including all the amyloidogenic proteins that aggregate extracellularly (Fig. 1). Secreted proteins are targeted to the ER cotranslationally by N-terminal targeting sequences, which are proteolytically removed upon entry into the ER lumen (Plate and Wiseman, 2017; Sun and Brodsky, 2019; Romine and Wiseman, 2020). In the ER, these proteins engage ATP-dependent ER chaperoning pathways, folding enzymes (e.g., protein disulfide isomerases [PDIs]), and lectin-based chaperones (e.g., calnexin/calreticulin) to facilitate their folding into native 3D conformations (Plate and Wiseman, 2017; Sun and Brodsky, 2019; Romine and Wiseman, 2020). Once folded, proteins are trafficked from the ER to the Golgi in a coat protein complex II (COPII)–dependent manner and subsequently directed to downstream secretory environments such as the extracellular space. Proteins unable to attain a folded conformation through interaction with ER folding pathways are instead recognized by ER-localized factors that direct these proteins to degradation by the proteasome via ER-associated degradation (ERAD) or the lysosome through autophagic mechanisms such as ER-phagy (Plate and Wiseman, 2017; Sun and Brodsky, 2019; Romine and Wiseman, 2020). The partitioning of proteins between ER folding and degradation is a process referred to as ER quality control and functions to promote secretion of folded functional proteins, while limiting the accumulation of nonnative, potentially aggregation-prone proteins in extracellular environments.

Despite the general efficacy of ER quality control, deficiencies in ER proteostasis induced by genetic, environmental, or aging-related stress can challenge extracellular environments. For example, ER quality control pathways are effective at limiting the secretion of highly destabilized, highly aggregation-prone variants of amyloidogenic proteins such as TTR and lysozyme, thus preventing their downstream pathologic aggregation (Sekijima et al., 2005; Kumita et al., 2006). However, more moderately destabilized, yet still aggregation-prone, variants of these proteins escape ER quality control and are efficiently secreted to the extracellular space, where they can aggregate into toxic oligomers and amyloid fibrils implicated in amyloid disease pathogenesis. Further, destabilized, aggregation-prone variants of TTR or the destabilized Z-variant of A1AT, implicated in A1AT deficiency, can be secreted in nonnative conformations that accumulate extracellularly as aggregates (Chen et al., 2016; Fra et al., 2016). While the specific conformation of these proteins secreted from mammalian cells can be difficult to define, these results show that destabilized proteins can be secreted as either aggregation-prone monomers/oligomers or aggregates, highlighting the sensitivity of destabilized disease-associated proteins to imbalances in ER quality control.

Pathologic insults that challenge ER proteostasis and quality control (i.e., ER stress) increase secretion of proteins in nonnative conformations that rapidly aggregate in extracellular environments. For example, in cell culture models, the secretion of the amyloidogenic protein TTR as native tetramers is reduced in response to ER stress (Chen et al., 2016). This increases the population of nonnative TTR in the extracellular environment, bypassing the rate-limiting tetramer dissociation step of TTR aggregation and accelerating aggregation into soluble TTR oligomers commonly associated with toxicity (Chen et al., 2016). This suggests that increased secretion of nontetrameric TTR from the liver—the primary site of TTR synthesis—could exacerbate protein aggregation in the blood and contribute to the increased population of nonnative TTR observed in plasma collected from TTR amyloid disease patients suffering from polyneuropathy (Schonhoft et al., 2017; Jiang et al., 2021). Similar results were observed in mouse models of TTR amyloid disease, where age-dependent deposition of TTR aggregates in the heart correlated with aberrant expression of proteostasis factors in the liver (Buxbaum et al., 2012). These results, and others discussed in previous reviews (Plate and Wiseman, 2017; Romine and Wiseman, 2020), highlight the important role of ER quality control in dictating both the amount and conformational integrity of proteins secreted into extracellular environments.

### Indirect regulation of extracellular proteostasis through the unfolded protein response (UPR)

**Figure 2.**
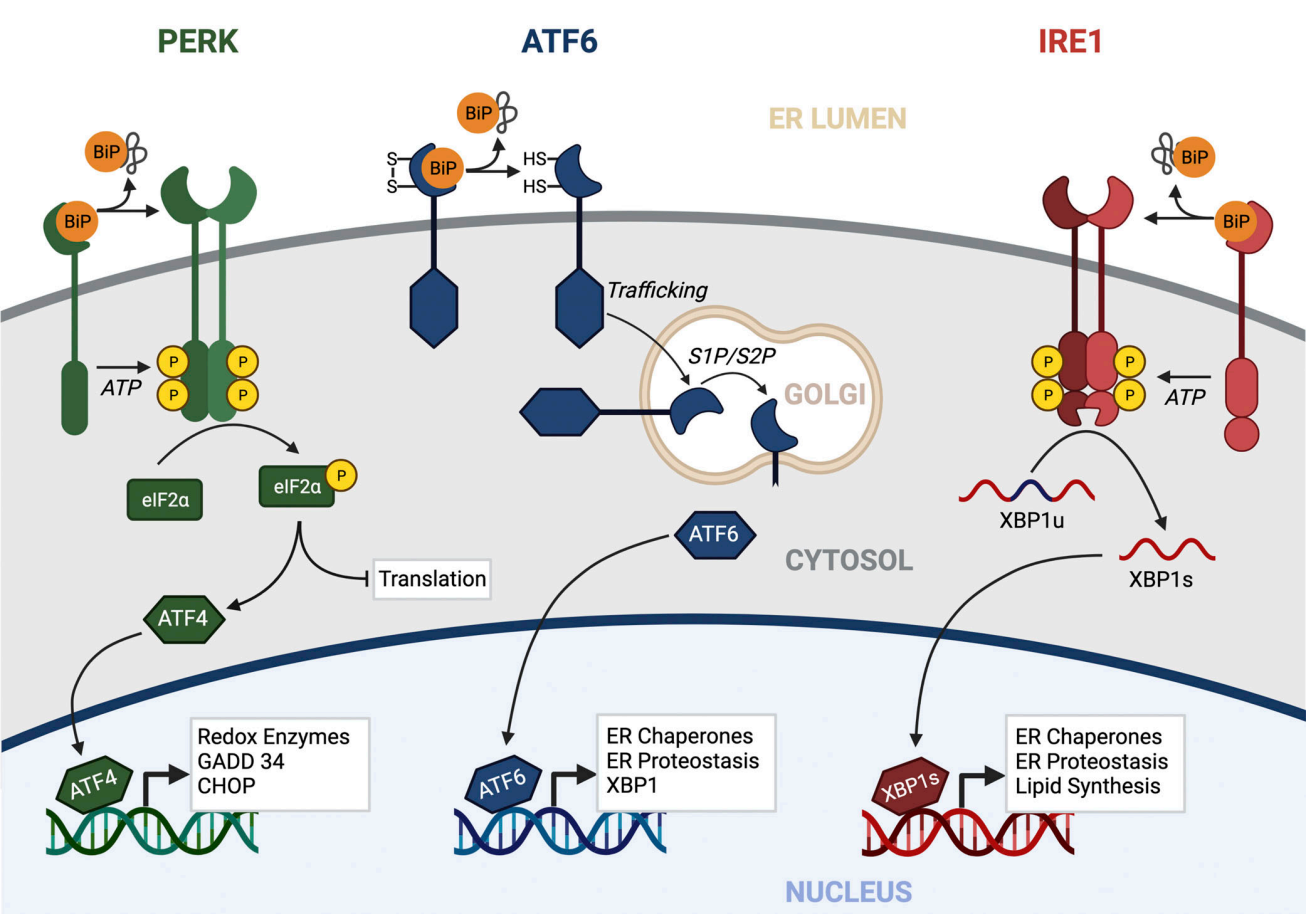
**The UPR.** The metazoan UPR comprises three integrated signaling pathways activated downstream of the ER membrane proteins PERK, ATF6, and IRE1. In response to ER stress, these pathways are activated through mechanisms including dissociation of the ER HSP70 chaperone BiP from their luminal domains. Once activated, UPR signaling promotes adaptive remodeling of ER proteostasis pathways and global cellular physiology through both a transient attenuation of new protein synthesis (downstream of PERK) and activation of the stress-responsive transcription factors ATF4, ATF6, and XBP1s (downstream of PERK, ATF6, and IRE1, respectively). Created with BioRender.com.

To protect extracellular proteostasis in response to ER stress, cells activate the UPR. The UPR comprises three integrated signaling pathways activated downstream of the ER stress–sensing transmembrane proteins IRE1, PERK, and ATF6 (Fig. 2). In response to imbalances in ER proteostasis, these pathways are activated through well-described mechanisms (Gardner et al., 2013; Karagoz et al., 2019; Hetz et al., 2020) to promote adaptive remodeling of the ER (and other biological pathways) through both transient attenuation of new protein synthesis (downstream of PERK) and activation of stress-responsive transcription factors including XBP1s (downstream of IRE1), ATF6 (a cleaved product of full-length ATF6), and ATF4 (downstream of PERK). Numerous reviews discuss the signaling mechanisms and functional implications of UPR activation (Gardner et al., 2013; Karagoz et al., 2019; Hetz et al., 2020). Here, we specifically focus on describing how these UPR pathways influence extracellular proteostasis by controlling the amount, stability, and conformation of the secreted proteome.

A primary function of the UPR is to promote adaptive remodeling of ER proteostasis pathways to mitigate ER stress and restore ER proteostasis. This is primarily achieved through the adaptive remodeling of ER quality control pathways involved in protein folding, trafficking, and degradation by the UPR-associated transcription factors XBP1s and ATF6 (Fig. 2). These transcription factors induce overlapping, but distinct, sets of ER proteostasis genes, indicating that they can differentially influence ER quality control and overall ER function (Shoulders et al., 2013). Consistent with this, genetic activation of XBP1s or ATF6 differentially impacts ER quality control of destabilized, aggregation-prone proteins. For example, selective ATF6 activation preferentially reduces secretion of destabilized, aggregation-prone variants of TTR, LC, and α-1-antitrypsin, without significantly impacting secretion of more stable, non–aggregation-prone variants of the same proteins (Smith et al., 2011; Chen et al., 2014; Cooley et al., 2014). In contrast, XBP1s overexpression reduces secretion of amyloidogenic Aβ through increased targeting of destabilized amyloid precursor protein (APP) variants to ER degradation pathways (Kaneko et al., 2010). These ATF6- and XBP1s-dependent alterations in the secretion of destabilized, aggregation-prone protein are likely attributed to the remodeling of ER quality control pathways afforded by these transcription factors. For example, ATF6-dependent reductions in amyloidogenic LC secretion correspond to increased interactions with ATF6-regulated ER chaperones (Plate et al., 2019). Alternatively, XBP1s-dependent expression of ERAD factors such as HRD1 is associated with increased APP degradation that limits secretion of amyloidogenic Aβ (Kaneko et al., 2010).

The extracellular aggregation of amyloidogenic proteins is concentration dependent. Thus, reducing secretion of destabilized, aggregation-prone proteins through ATF6- or XBP1s-dependent remodeling of ER quality control pathways should reduce extracellular aggregation of these proteins into toxic oligomers and amyloid fibrils. Consistent with this, genetically increasing ATF6 or XBP1 activity in cell culture models reduces extracellular aggregates comprising amyloidogenic proteins (Chen et al., 2014; Cooley et al., 2014). Thus, the transcriptional regulation of ER quality control through IRE1/XBP1s or ATF6 activation can indirectly influence extracellular proteostasis by controlling the amounts of aggregation-prone proteins in the extracellular environment.

The PERK arm of the UPR also has a key role in regulating ER quality control and extracellular proteostasis, especially during conditions of ER stress. Genetic or pharmacologic inhibition of PERK decreases trafficking and increases ER stress–dependent accumulation of numerous secretory proteins including WT proinsulin, WT collagen, and mutant rhodopsin in mammalian cells (Harding et al., 2012; Athanasiou et al., 2017; Hisanaga et al., 2018; Sowers et al., 2018). PERK deficiency also impairs the ability of ER quality to regulate extracellular proteostasis during ER stress. Pharmacologic inhibition of PERK in cell culture models enhances ER stress–dependent secretion of TTR in nonnative conformations and increases the relative accumulation of soluble TTR aggregates in conditioned media (Romine and Wiseman, 2019). While the specific contributions of PERK-dependent translational attenuation and transcriptional signaling in the regulation of ER quality control and extracellular proteostasis remain to be defined, these results highlight an important role for PERK in regulating both secretory proteostasis and the integrity of the secreted proteome.

### Imbalanced UPR signaling in amyloid disease pathogenesis

The above results demonstrate that UPR-dependent regulation of ER quality control indirectly influences extracellular proteostasis by controlling the amounts of destabilized, aggregation-prone proteins secreted into extracellular environments. This suggests that imbalances in UPR signaling could directly contribute to the pathogenesis of protein aggregation diseases such as amyloid disorders. Consistent with this, alterations in UPR signaling are implicated in the onset and pathogenesis of numerous amyloid diseases. For example, chronic activation of PERK is implicated in the neurodegeneration associated with amyloid diseases including AD and the prionoses (Bell et al., 2016; Hughes and Mallucci, 2019). The endogenous expression of disease-associated, amyloidogenic TTR variants in induced pluripotent stem cell–derived hepatocytes increase expression of ATF6 and XBP1s target genes, suggesting an important role for UPR-dependent regulation of secretory proteostasis in TTR amyloid disease (Giadone et al., 2020). Similar results were observed in cells expressing amyloidogenic variants of lysozyme (Kamada et al., 2015). UPR signaling, like many other stress-responsive signaling pathways such as the heat shock response, is also dysregulated during aging (Kaushik and Cuervo, 2015; Frakes and Dillin, 2017; Gerakis and Hetz, 2018; Taylor and Hetz, 2020), a known risk factor for many amyloid diseases.

While the specific impact of altered UPR signaling on the extracellular aggregation of amyloidogenic proteins in human disease remains an open question, these results, in combination with those showing the importance of UPR-dependent regulation of ER quality control on extracellular proteostasis, strongly suggest that imbalanced UPR activity contributes to the extracellular protein aggregation implicated in amyloid disease pathogenesis. One piece of evidence potentially supporting this assertion is from a mouse model of TTR amyloidosis in which age-dependent deposition of TTR aggregates in the heart correlate with altered expression of some UPR target genes in the liver, the primary site of TTR synthesis (Buxbaum et al., 2012). This suggests that impaired UPR-dependent regulation of ER quality control in the liver contributes to the increased extracellular aggregation and deposition of TTR, potentially through mechanisms including increased hepatic secretion of TTR in nonnative, aggregation-prone conformations. Further defining the contribution of dysregulated ER quality control regulation in tissues secreting amyloidogenic proteins to their pathologic extracellular aggregation will have profound implications in our understanding of the pathogenesis of amyloid diseases, which, in the past, were considered diseases of the tissue where amyloid was deposited, not where the amyloidogenic proteins were synthesized.

## Regulating extracellular chaperoning pathways during stress

### Extracellular proteostasis is regulated by secreted chaperones

Once secreted, proteins are subject to harsh conditions of the extracellular environment that challenge their ability to maintain a folded, functional conformation. To protect the integrity of the secreted proteome under these conditions, metazoans evolved a network of secreted chaperones that can bind nonnative proteins in extracellular environments to both prevent protein misfolding and/or aggregation and promote the targeting of nonnative proteins to tissues/cells (e.g., liver and macrophages) that mediate their degradation through endolysosomal pathways (Fig. 1; Lai and McLaurin, 2012; Heckmann et al., 2019). Unlike intracellular chaperones, extracellular chaperones are generally ATP independent, allowing them to function in the low-ATP environment of the blood and CSF (Wyatt et al., 2013). Extracellular chaperones are primarily defined by three criteria: (1) their ability to be readily secreted from mammalian cells, primarily owing to the lack of an ER retention motif, (2) their ability to bind nonnative proteins, and (3) their ability to prevent protein aggregation in vitro. In vivo, extracellular chaperones are often found codeposited with amyloid fibrils in human tissues. This codeposition of extracellular chaperones could reflect deficiencies in extracellular chaperoning activity, due to low plasma concentrations or damage to the extracellular chaperone that limits their ability to inhibit aggregation of amyloidogenic proteins. In this case, the extracellular chaperones would be able to bind mature fibrils but unable to prevent fibrillogenesis. Alternatively, extracellular chaperones may enhance aggregation and fibrillogenesis as a mechanism to prevent accumulation of toxic oligomeric structures. For example, in vitro studies indicate that TTR diverts the Aβ precursor and its oligomers into amorphous, nontoxic aggregates (Cascella et al., 2013). Further, codeposition could also reflect a protective mechanism involved in stabilizing the deposited fibrils and preventing their resolubilization to the potentially toxic oligomers that are more often associated with tissue damage.

Currently, several extracellular chaperones have been identified using the above criteria (Table 1). These include the canonical extracellular chaperone clusterin, haptoglobin, α2 macroglobulin, αB crystallin, casein, and more recently, neuroserpin (Dabbs et al., 2013; Wyatt et al., 2013; Chaplot et al., 2020; Satapathy and Wilson, 2021a; West et al., 2021). While some of these secreted chaperones appear to have relatively narrow substrate specificities in vivo (e.g., haptoglobin for hemoglobin, α_2_ macroglobulin for β_2_ microglobulin), others such as clusterin promiscuously bind and inhibit aggregation of multiple different proteins (Itakura et al., 2020; Satapathy and Wilson, 2021b). An additional set of proteins has also been described as displaying extracellular chaperoning activity in neurodegenerative diseases. These include progranulins, S100A proteins, BRICHOS domain–containing proteins, ProSAAS, 7B2, and HSPB1 (Table 1); however, it remains somewhat unclear if these molecules function systemically or only in the local environment upon release from neurons or glia. Further, the in vivo specificity of these chaperones for substrates beyond those associated with neurodegenerative disease (e.g., Aβ) remains to be further defined, although many have been shown to broadly inhibit aggregation of proteins in vitro (Satapathy and Wilson, 2021a). Regardless, it is becoming increasingly clear that a number of secreted chaperones contribute to protecting the extracellular proteome from pathologic misfolding and/or aggregation.

**Table 1.** List of extracellular chaperones and their substrate specificity and regulation

Extracellular chaperone	Gene	In vivo substrate specificity	Regulation during stress
7B2	*SCG5*	Aβ in AD plaques; α-synuclein in Lewy bodies	Promoter contains heat shock element–like sequences but function not explored (Braks et al., 1996)
α_2_-Macroglobulin	*A2M*	β_2_-Microglobulin	Induced by the acute phase response (Ehlting et al., 2021)
αB crystallin	*CRYAB*	Broad intracellular substrate specific	Induced by the heat shock response; normally an intracellular protein (Ryno et al., 2014)
β-Casein	*CSN2*	Milk proteins, amyloid, and nonamyloid aggregates	Induced in mammary epithelial cells by lactogenic hormones; heat stressing cultured mammary cells increases production via the UPR (Mizusawa et al., 2019)
Brichos-domain containing proteins	*BRI1*, *BRI2*	Intramolecular in pulmonary surfactant C, AD plaques	Undefined
Clusterin	*CLU*	Broad substrate specificity	Induced by the heat shock response and oxidative stress; relocalized to the cytosol during ER stress (Nizard et al., 2007; Li et al., 2013; Niforou et al., 2014)
ERdj3/DNAJB11	*DNAJB11*	Broad substrate specificity	Induced by the UPR; increased secretion during ER stress (Genereux et al., 2015)
Haptoglobin	*HP*	Hemoglobin	Induced by the acute phase response (Ehlting et al., 2021)
HSPB1	*HSPB1*	Mutations associated with hereditary neuropathies, associated with Tau in tauopathies	Induced by the heat shock response; normally an intracellular protein (Ryno et al., 2014)
Neuroserpin	*SERPINI1*	Amyloid plaques in AD	Undefined
Progranulin	*GRN*	Cathepsin D, β-glucocerebrosidase	Induced during hypoxia and exercise (Wang et al., 2021)
ProSAAS	*PCSK1N*	Neurodegenerative diseases, AD, Parkinson’s, Pick’s	Induced during ER and heat stress; secretion is reduced under these conditions (Shakya et al., 2020)
S100A proteins	*S100A1-A16*	Aβ in plaques; breadth not clear	Induced in response to diverse inflammatory and oxidative insults (Gonzalez et al., 2020)
Transthyretin	*TTR*	Amyloid fibrillogenesis	Induced by the heat shock response in brain, but not liver (Wang et al., 2014)

Surprisingly, some amyloidogenic proteins such as TTR have also demonstrated activities consistent with extracellular chaperones (Table 1). TTR inhibits aggregation of disease-associated secreted proteins including Aβ at substoichiometric levels (Buxbaum and Johansson, 2017; Cao et al., 2020). The ability of TTR to inhibit aggregation of amyloidogenic substrates such as HypF-N is comparable to that observed for other secreted chaperones (e.g., clusterin), highlighting the potential for TTR to serve as an extracellular chaperone. Like clusterin and other secreted chaperones (Chaplot et al., 2020), TTR colocalizes with Aβ in human AD plaques, further indicating its possible role as an extracellular chaperone (Buxbaum and Johansson, 2017; Cao et al., 2020; Ciccone et al., 2020). Supporting this notion, acceleration of Aβ aggregation was observed in an AD mouse model given an anti-TTR antibody and in mice in which the endogenous *Ttr* gene was silenced by targeted interruption, while overexpression of WT human TTR inhibited the pathologic and behavioral changes usually seen in these mice (Li et al., 2011).

With the identification of secreted proteins such as TTR that have chaperoning activity, it is likely that we are just beginning to understand the scope and activity of the extracellular chaperoning network. Many good reviews discuss in greater detail our current understanding of the disease relevance of extracellular chaperones and the mechanisms by which secreted chaperones regulate extracellular proteostasis (e.g., prevention of toxic protein aggregation, receptor-mediated targeting for degradation; Wyatt et al., 2012; Dabbs et al., 2013; Wyatt et al., 2013; Chaplot et al., 2020;Satapathy and Wilson, 2021b). We direct the reader to these reviews for additional information on these topics. Here, we instead focus on the regulation of secreted chaperones and their impact on extracellular proteostasis in response to pathologic insult.

### Regulation of extracellular chaperones during ER stress

Despite the importance of extracellular chaperones in extracellular proteostasis maintenance, little is known about their regulation in response to pathologic insults. Interestingly, while ER stress can increase secretion of proteins in nonnative conformations, the secretion of the prominent extracellular chaperone clusterin is reduced in cells cultured under these conditions (Nizard et al., 2007; Li et al., 2013). This is mediated through the retro-translocation of clusterin from the ER to the cytosol, where it is thought to protect through mechanisms including increased targeting of secretory proteins for proteasomal or autophagic degradation (Nizard et al., 2007; Materia et al., 2012; Li et al., 2013; Zhang et al., 2014; Satapathy and Wilson, 2021a). However, the increased intracellular localization of clusterin reduces the population of secreted clusterin that can protect extracellular environments from ER stress–dependent increases in the secretion of nonnative proteins.

**Figure 3.**
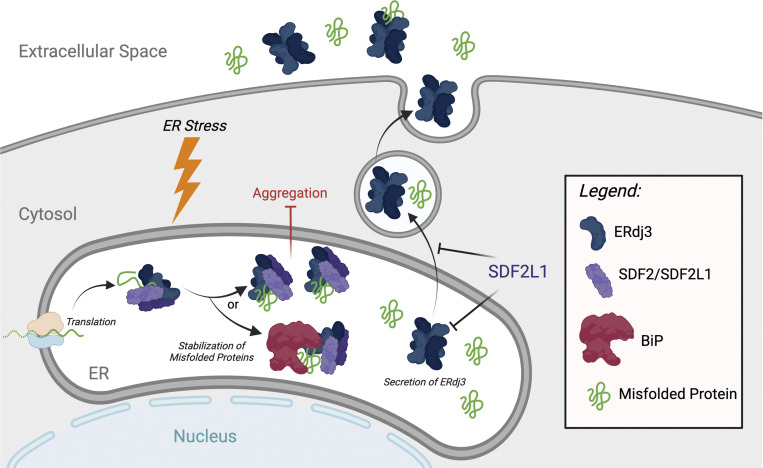
**ERdj3 is a UPR-regulated secreted chaperone.** In the absence of ER stress, ERdj3 dimers engage ER-localized SDF2/SDF2L1 to bind misfolded proteins and deliver them to BiP for ATP-dependent chaperoning. Upon ER stress, ERdj3 assembles into a homotetramer that is secreted to the extracellular space, where it can function as an extracellular chaperone. Created with BioRender.com.

In contrast to clusterin, the UPR-regulated chaperone ERdj3/DNAJB11 is secreted during conditions of ER stress (Genereux et al., 2015). ERdj3 is an HSP40-type cochaperone that functions in the ER as part of the ATP-dependent binding immunoglobulin protein (BiP) HSP70 pathway, where it recruits nonnative proteins to BiP and stimulates BiP ATPase activity (Pobre et al., 2019). In the ER, ERdj3 primarily functions to promote the proper folding of secretory proteins (Pobre et al., 2019); however, ERdj3 has also been implicated in the targeting of specific substrates such as β-glucocerebrosidase to degradation via ERAD (Tan et al., 2014). Like many other components of the BiP chaperoning pathway, *ERDJ3/DNAJB11* is transcriptionally regulated during conditions of ER stress downstream of IRE1/XBP1s and ATF6 (Genereux et al., 2015). However, unlike many other ER proteostasis factors, ERdj3 lacks a canonical C-terminal KDEL ER retention motif. Instead, ERdj3 is retained in the ER through its interactions with the ER-resident proteins BiP and SDF2/SDF2L1, the latter forming heterotetrameric complexes with ERdj3 that retain ERdj3 in the ER (Fig. 3; Genereux et al., 2015; Hanafusa et al., 2019). During conditions of ER stress, ERdj3 assembles into homotetrameric complexes that are unable to be retained within the ER, allowing efficient secretion to extracellular environments (Genereux et al., 2015; Chen et al., 2017). While the underlying molecular mechanisms responsible for the increased assembly of ERdj3 homotetramers are not fully understood, it may reflect reduced availability of BiP or alterations in the stability or assembly of SDF2/SDF2L1 that limits the potential interaction between these proteins and ERdj3 (Genereux et al., 2015; Hanafusa et al., 2019).

ER stress–dependent increases in ERdj3 secretion could impact extracellular proteostasis in two distinct ways. First, secreted ERdj3 can function as a canonical extracellular chaperone, preventing the aggregation of proteins in extracellular environments (Fig. 3), evident by the ability of ERdj3 to limit Aβ aggregation in vitro at substoichiometric concentrations and prion toxicity in cell culture models (Genereux et al., 2015). Alternatively, ERdj3 can also be cosecreted with destabilized, aggregation-prone proteins including amyloidogenic TTR and APP, preemptively protecting the extracellular environment from their increased secretion (Genereux et al., 2015). This cosecretion of ERdj3-substrate complexes is regulated by BiP availability. Under normal conditions, ERdj3 delivers substrates to BiP for ATP-dependent chaperoning; however, when BiP is limiting, such as during ER stress, ERdj3 is unable to deliver substrates to BiP and is instead cosecreted in complex with substrates. Through this mechanism, UPR-dependent regulation of ERdj3 secretion links ER and extracellular chaperoning capacity to help protect the extracellular environment during conditions of ER stress.

ER stress–dependent increases in ERdj3 secretion could also serve other functions, independent of proteostasis regulation. For example, secretion of the fly ERdj3 homolog Shriveled is involved in maintaining the stem cell niche through integrin signaling, suggesting potential receptor-mediated signaling activities for ERdj3 (Lee et al., 2016). Similarly, ERdj3 binds proteogenin to both suppress neurogenesis and regulate craniofacial development in vivo (Wong et al., 2010; Wang et al., 2013). While the potential for secreted ERdj3 to similarly regulate signaling pathways during ER stress remains to be defined, these results highlight the potential for stress-dependent increases in secreted ERdj3 to function through nonchaperoning activity to regulate organismal physiology. Interestingly, secreted ERdj3 has also been identified as a potential biomarker for nephrotic syndrome, suggesting potential diagnostic benefits for ER stress–dependent increases in ERdj3 secretion (Tousson-Abouelazm et al., 2020; Li and Chen, 2021).

Other ER chaperones and folding factors can also be trafficked to extracellular environments during ER stress, although the specific benefit of this secretion on extracellular proteostasis regulation is not clear. For example, the ATP-dependent ER chaperone BiP can be trafficked to the cell surface or secreted; however, these populations of BiP appear to primarily regulate other aspects of cellular physiology including proliferation, inflammation, and immunological signaling, as opposed to extracellular proteostasis regulation (Lee, 2014; Zhu and Lee, 2015; Meyer and Doroudgar, 2020). Alternatively, PDIs including PDIA1, PDIA3, and PDIA6 are trafficked to the cell surface, where they regulate disulfides in membrane receptors to influence biological processes including coagulation, integrin signaling, and viral entry (Lahav et al., 2000; Cho et al., 2008; Swiatkowska et al., 2008; Kohli et al., 2021). Further, mesencephalic astrocyte-derived neurotrophic factor (MANF), a protein that possesses chaperone activity and is involved in regulating ATP-dependent BiP chaperoning in the ER, is secreted to extracellular environments during ER stress (Jӓntti and Harvey, 2020; Meyer and Doroudgar, 2020). While the specific impact of secreted MANF on extracellular proteostasis regulation remains to be established, it is clear that exogenous administration of MANF is protective against pathologic insults such as ischemia/reperfusion (Glembotski et al., 2012; Yang et al., 2014). These results demonstrate that, apart from ERdj3, UPR-dependent regulation of other secreted proteins has the potential to influence extracellular proteostasis during conditions of stress; however, the specific benefits of ER stress–dependent increases in these secreted chaperones on extracellular proteostasis regulation remain to be further established.

### Regulating extracellular chaperoning pathways in response to other insults

Apart from ER stress, other types of pathologic insults could also challenge extracellular proteostasis. Increased temperature or oxidative stress can promote misfolding and/or aggregation of secreted proteins in vitro and in cell culture models. This suggests that increased core body temperature seen in the inflammatory febrile response to infection or increases in oxidative stress that can lead to oxidative protein damage could similarly promote misfolding of secreted proteins. Consistent with this, aging-associated oxidative modifications of amyloidogenic proteins such as TTR can destabilize the native protein and promote cytotoxic aggregation into toxic oligomers and amyloid fibrils, indicating a potential role for oxidative stress in TTR amyloid disease pathogenesis (Zhao et al., 2013). Despite this, the contributions of stress-responsive signaling pathways in regulating the composition and activity of secreted chaperone networks in response to non-ER stress insults remain poorly understood. Clusterin is induced by many different types of cellular stress, including heat stress and oxidative stress, through the activity of transcription factors including HSF1 (Niforou et al., 2014), the master regulator of the cytosolic heat shock response (Magalhães and Saraiva, 2012; Vihervaara and Sistonen, 2014). However, the impact of this regulation on clusterin extracellular chaperoning activity is unclear. As observed during ER stress, heat reduces clusterin secretion and increases accumulation of clusterin in other intracellular environments such as the cytosol (Magalhães and Saraiva, 2012). Thus, HSF1-dependent regulation of clusterin may function more as mechanism to promote intracellular proteostasis in response to cytosolic insults.

TTR is also transcriptionally regulated by HSF1 in the brain, although not in other tissues such as the liver (Wang et al., 2014). Neuronal HSF1-dependent induction of TTR increases its synthesis and secretion, suggesting a potential role for secreted TTR in regulating extracellular proteostasis during stress. While TTR and clusterin are both secreted proteins and regulated by HSF1, they differ considerably in their ability to inhibit the potentially pathogenic aggregation of proteins. In addition to its ability to inhibit aggregation into amyloid fibrils, clusterin is capable of inhibiting aggregation of structurally diverse proteins induced by varied insults including heat and chemical denaturation (Wyatt et al., 2011; Gregory et al., 2017; Itakura et al., 2020). In contrast, TTR chaperoning activity is relatively specific for inhibiting amyloidogenesis, regardless of the precursor protein. For example, TTR inhibits in vitro fibril formation of human amyloidogenic proteins like Aβ (Schwarzman et al., 1994; Li et al., 2011; Nilsson et al., 2018), as well as the functional bacterial amyloids involved in biofilm formation (e.g., CsgA; Jain et al., 2017), while showing no appreciable effect on less structured aggregates produced in response to other models of aggregation (West et al., 2021). These differential substrate specificities suggest clusterin and TTR may serve distinct roles in regulating extracellular proteostasis during conditions of stress, although this remains to be defined.

### Discovering new stress-responsive mechanisms involved in regulating extracellular proteostasis

As discussed above, we still have much to learn regarding stress-responsive regulation of extracellular chaperoning pathways, necessitating the development of new approaches to identify signaling pathways and secreted chaperones that regulate extracellular proteostasis. To address this, the David lab recently developed a *Caenorhabditis elegans* based approach to identify stress-regulated factors that promote extracellular proteostasis during pathogenic attack (Gallotta et al., 2020). Using fluorescently tagged aggregation-prone proteins, they performed an RNAi screen to identify secreted proteins whose depletion exacerbated extracellular protein aggregation. Using this approach, they identified several extracellular regulators of proteostasis that are transcriptionally induced as part of the innate immune response (Gallotta et al., 2020). Intriguingly, overexpression of these proteins improves organismal survival in response to pathogenic attack, indicating that their increased expression enhances host defense. While the specific functional impact of increased expression of these extracellular proteostasis regulators during pathogenic attack remains to be further defined, one intriguing idea is that increasing extracellular proteostasis capacity in this way could prevent the potential aggregation of antimicrobial peptides that are secreted under these conditions.

This study, which is one of the first to directly screen for regulators of extracellular proteostasis, highlights new opportunities to understand the critical role for stress-responsive regulation of extracellular proteostasis in response to pathologic insults and paves the way for similar approaches to be applied for mapping the regulation of extracellular chaperoning networks in other models. While the conservation of the pathogen-stimulated increase in extracellular proteostasis capacity observed in worms remains to be established, mammalian homologs of many extracellular regulators identified in this study, including amyloid-like protein 2 (APLP2) and the brevican and neurocan core proteins, were found to be altered in mass spectrometric analysis of CSF from AD patients (Khoonsari et al., 2016; Gallotta et al., 2020), suggesting that these proteins could similarly influence extracellular proteostasis in the pathogenesis of AD and potentially other neurodegenerative diseases (Muller et al., 2017; Rahman and Lendel, 2021). However, this remains to be established.

Other approaches have also been applied to identify regulators of extracellular proteostasis. One such strategy used misfolded protein “baits” to identify human serum proteins that show chaperoning activity (Geraghty et al., 2021). Through these efforts, proteins such as vitronectin and plasminogen activator 3 were identified as potential extracellular chaperones that could inhibit aggregation of aggregation-prone proteins including amyloidogenic Aβ and denatured citrate synthase, the latter serving as a model of amorphous aggregation. While the specific importance of these proteins in regulating extracellular proteostasis in vivo or in response to specific cellular insults remains to be established, these types of strategies open the door to identify new components of extracellular proteostasis networks that could be actively regulated to protect the secreted proteome in response to different types of stress.

## Therapeutic targeting of stress-responsive signaling to regulate extracellular proteostasis

The potential to mitigate extracellular aggregation by targeting stress-responsive signaling pathways that regulate the secreted proteome is an attractive strategy to attenuate pathology associated with etiologically diverse protein aggregation diseases. Numerous compounds have been identified that activate the ATF6 and IRE1/XBP1s signaling arms of the UPR responsible for regulating ER and secretory proteostasis environments. These compounds provide opportunities to reduce secretion and/or extracellular aggregation of amyloidogenic proteins implicated in disease pathogenesis. One such compound, AA147, activates ATF6-dependent ER remodeling through a mechanism involving compound metabolic activation and covalent modification of PDIs involved in regulating ATF6 signaling (Plate et al., 2016; Paxman et al., 2018). Interestingly, AA147 selectively reduces secretion of destabilized, aggregation-prone variants of amyloidogenic proteins such as TTR and LC to mitigate their concentration-dependent aggregation in extracellular environments (Plate et al., 2016). However, AA147 does not influence secretion of stable, nonamyloidogenic variants of these proteins or the endogenous secretory proteome. These results mimic those observed with genetic ATF6 activation, although, at least in the context of AA147-dependent reductions in amyloidogenic LC secretion, the benefits may result from upstream PDI modification and not ATF6 activation (Rius et al., 2021). Pharmacologic IRE1/XBP1s activation also limits secretion of amyloidogenic, aggregation-prone Aβ through a mechanism involving increased partitioning of APP to ERAD (Grandjean et al., 2020), again mimicking results observed upon genetic XBP1s overexpression (Kaneko et al., 2010). While these results highlight the potential for pharmacologically targeting adaptive aspects of UPR signaling using small molecules to mitigate imbalances in extracellular proteostasis implicated in a broad range of etiologically diverse diseases, more work is required to fully define the therapeutic potential for this approach to limit the pathologic extracellular aggregation of proteins associated with human disease.

Apart from the UPR, pharmacologic approaches to activate other stress-responsive pathways involved in regulating extracellular proteostasis, such as the HSF1-regulated heat shock response, also offer opportunities to improve extracellular proteostasis in disease. Numerous HSF1-activating compounds have been identified to activate this pathway through multiple different mechanisms (Neef et al., 2010; Calamini et al., 2011; Neef et al., 2011; Carpenter and Gökmen-Polar, 2019). Intriguingly, compounds such as the HSF1 activator celastrol and iso-α acids increase *Ttr* transcription and secretion in the brain, suggesting the potential for increasing TTR extracellular chaperoning activity in the central nervous system (Wang et al., 2014; Fukuda et al., 2018). However, the benefit of HSF1-dependent regulation of TTR or other extracellular chaperones in protecting extracellular environments during pathologic insults remain to be established.

As we learn more about the key role for stress-responsive pathways involved in regulating extracellular proteostasis, new opportunities will emerge to identify strategies to target these pathways to mitigate pathologic imbalances in extracellular proteostasis implicated in human disease. However, a key challenge in defining the specific contributions of stress-responsive remodeling of extracellular proteostasis in disease outcomes is separating the impact of specific treatments on intracellular versus extracellular pathways. Activating these types of stress signaling pathways impacts environments both inside and outside of the cell, making it difficult to ascribe specific benefits that directly improve extracellular proteostasis. While genetic depletion of key secreted chaperones (e.g., HSF1-regulated TTR in the brain) offers a way to define these types of contributions, more information regarding the molecular mechanisms responsible for stress-responsive regulation of extracellular proteostasis is required to truly define the benefits of specific therapeutic approaches in mitigating pathologic imbalances of extracellular proteostasis in the context of amyloid diseases or other diseases associated with the misfolding and/or aggregation of secreted proteins.

## Concluding remarks and future perspectives

Despite the importance of extracellular proteostasis in health and disease, we are only beginning to understand the molecular mechanisms responsible for regulating the integrity of the secreted proteome. However, it is already clear that this regulation is critical for preventing imbalances in extracellular proteostasis and that the failure of these mechanisms contributes to the pathologic aggregation of proteins implicated in etiologically diverse diseases including many amyloid diseases. As we learn more of the stress-responsive mechanisms responsible for regulating extracellular proteostasis, we will reveal new insights into the importance of ER and extracellular proteostasis pathways, and more specifically their regulation, in protecting the extracellular environment from the accumulation of nonnative, aggregation-prone proteins. This will likely identify new ways in which challenges to extracellular proteostasis contribute to the pathogenesis of diverse diseases (e.g., increases in nonnative, nonfunctional secreted proteins that promote loss-of-function phenotypes). Similarly, an improved understanding of how other mechanisms such as the unconventional secretion and extracellular accumulation of aggregation-prone proteins like tau or α-synuclein (Goedert et al., 2017; Uemura et al., 2020) influence extracellular proteostasis will also likely reveal additional ways in which pathologic insults challenge the extracellular proteostasis network. Further, identifying new stress-responsive mechanisms responsible for regulating extracellular proteostasis will define novel therapeutic opportunities to mitigate pathologic imbalances in extracellular proteostasis. Thus, while we are still at the beginning of this area of biology, the emerging data offer significant opportunities to gain new insights into how organisms integrate intra- and extracellular proteostasis pathways to protect the secreted proteome during conditions of stress.
